# Cancer Cell Models for the Development of Anti-Cancer Drugs

**DOI:** 10.3390/ijms232214457

**Published:** 2022-11-21

**Authors:** Nitin T. Telang

**Affiliations:** Cancer Prevention Research Program, Palindrome Liaisons Consultants, Montvale, NJ 07646-1559, USA; ntelang3@gmail.com

In the multi-factorial etiology of organ-site cancers by suspect human chemical carcinogens, oncogenic virus, activation of RAS, Myc and HER-2 oncogenes, inactivation of TP53, RB and APC tumor suppressor genes represent early-occurring genetic events. Additionally, the multi-step processes of initiation, promotion and progression play critical roles in the development of the disease. Cancer initiation is induced by genotoxic etiological agents, promotion of initiated cells is induced by several epigenetic agents, and carcinogenesis of putative pre-neoplastic cells represents the process of progression. For example, in breast cancer biology, etiological agents induce lactogenic hormone-independent hyperplastic alveolar nodules in vivo or nodule-like alveolar lesions in vitro. Tumor development by transplantation of these lesions documents their pre-neoplastic nature [1]. Similarly, aberrant crypt foci represent putative pre-neoplastic lesions in colon cancer. Thus, these pre-neoplastic lesions represent specific and sensitive in vitro surrogate endpoint markers for carcinogenicity of chemicals, as well as for their experimental modulation.

The objective of this Special Issue was to provide a platform for multi-disciplinary contributions that utilize tissue culture models including, but not restricted to, organ culture, cell culture, ex vivo tumor explants, patient-derived tumor xeno-transplants, patient-derived tumor organoids and cancer stem cells. These approaches can be applied to conduct mechanistic studies that may lead to development of new anti-cancer drugs. Such in vitro studies, supported by quantifiable pathway specific molecular end-point biomarkers, may validate valuable experimental approaches to identify new drugs that may function as efficacious anti-cancer drugs and to prioritize these agents for their clinical relevance and translatability.

It was most gratifying to note enthusiastic response from all the contributors for submitting manuscripts containing excellent research in multi-disciplinary areas relevant to novel anti-cancer drugs and innovative methodology for drug delivery. Specifically, experimental approaches utilizing 3D bio-printing [2,3], liposome-mediated drug delivery [4], functional significance of estrogen receptors in initial events relevant to breast cancer development [5] and the development of models for drug-resistant colon cancer stem cells [6] provide strong evidence for innovative application of state-of-the art technology.

Published manuscripts included: 3D models relevant to tumor micro-environment; high throughput 3D models for multi-parametric quantitation of cancer; 3D bio-printing for cancer modelling; models for esophageal carcinoma; models for targeting tumor-associated macrophages in melanoma; improved liposomes to develop mitochondrial targeting drug delivery systems; a mammary gland organ culture model to investigate the role of estrogen receptor isoforms in breast carcinogenesis; models to evaluate CAR T cell function in preclinical cancer models; and the application of drug-resistant stem cells to identify testable alternatives for stem cell-targeted therapy of colon cancer.

All the peer-reviewed publications provided ample evidence for scientifically robust conceptual and technical aspects, well-designed experimental approaches, and excellent interpretation and discussion of the data. It has been a distinct pleasure and an honor to serve as the guest editor for this Special Issue.

## Data Availability

Not applicable.
